# Strategic donor behaviour and country vulnerability in health aid transitions

**DOI:** 10.1136/bmjgh-2023-012953

**Published:** 2023-11-08

**Authors:** Wenhui Mao, Kaci Kennedy McDade, Osondu Ogbuoji, Gavin Yamey, Sarah Blodgett Bermeo

**Affiliations:** 1 Center for Policy Impact in Global Health, Duke Global Health Institute, Durham, North Carolina, USA; 2 Margolis Center for Health Policy, Duke University, Washington, DC, USA; 3 Sanford School of Public Policy, Duke University, Durham, North Carolina, USA

**Keywords:** health economics, health policy

## Abstract

**Background:**

When countries reach the middle-income threshold, many multilateral donors, including Gavi, the Vaccine Alliance (Gavi), begin to withdraw their official development assistance (ODA), known as graduation. We hypothesised that bilateral donors might follow Gavi’s lead, except in countries where they have strategic interests. We aim to understand how bilateral donors behave after a recipient country graduates from Gavi support and how bilateral donors might treat Gavi support countries differently, based on ‘strategic interest’. We also aim to identify countries that were more vulnerable to ‘simultaneous’ transitions and financial cliffs after Gavi transition.

**Methods:**

This is an observational dyadic analysis using longitudinal data. We collected country-level data on 77 Gavi-eligible countries between 2009 and 2018 and paired donor and recipient country in a specific year to conduct dyadic analysis. We included Gavi graduation status and Gavi disbursement as explanatory variables. We controlled for (1) donor–recipient relationship variables that represent potential strategic relationships (eg, distance between donor and recipient country) and (2) recipient-level characteristics (eg, population, income). We used Odinary Least Squares regression, Tobit and two-part model in Stata SE 15.0.

**Findings:**

We found a country would receive $3.1 million less all sector ODA from a bilateral donor, and $0.6 million less health ODA, after they graduate from Gavi. For every additional 1% ODA a country would receive from Gavi, it would receive 0.14% more ODA and 0.16% more health ODA from individual bilateral donors. Gavi’s graduation status or disbursement brought more change in percentage term to health ODA than to total ODA. Additionally, Gavi’s graduation was observed to have a larger negative impact on bilateral ODA in the longer term. Countries that sent more migrants, had been colonised, and received more US military assistance tended to receive more ODA. There are similarities and differences across different donors and bilateral donors tend to provide more ODA to nearby countries and countries receiving fewer exports from the donor. We found that former colonies did not see a decline in aid after Gavi graduation.

**Conclusion:**

Bilateral donors behave in a similar manner to Gavi when it comes to funding health systems in low and middle-income countries. Therefore, some countries may be at risk of losing donor resources for health from a multitude of sources around the same time. However, countries that have a strategic interest in bilateral donors may be spared from such funding cliffs. This research has important implications for global health donors’ funding policies and approaches in addition to recipient countries’ transition planning.

**WHAT IS ALREADY KNOWN ON THIS TOPIC**

- Loss of external funding from a variety of sources can make it difficult to fill financial gaps with domestic resources. Multilateral donors tend to have more explicit graduation policies than bilateral donors. For example, Gavi, the Vaccine Alliance (Gavi), has a clear graduation approach and policy, informed by need-driven indicators such as gross domestic product per capita and vaccine coverage rates. Meanwhile, a recent study shows that bilateral donors behave strategically. It is unclear if there is interaction between bilateral and multilateral donors.

**WHAT THIS STUDY ADDS**

- This study found bilateral donors behave in a similar manner to Gavi when it comes to funding health systems in low and middle-income countries. A country would receive $3.1 million less all sector official development assistance (ODA) on average from a bilateral donor, and $0.6 million less health ODA, after they graduate from Gavi than prior to graduation. Assuming all other factors remain the same, for every additional 1% a country would receive from Gavi, it would receive 0.14% more ODA and 0.16% more health ODA from individual bilateral donors. Gavi’s transition was observed to have a larger negative impact on bilateral ODA in the longer term. We found that former colonies did not see a decline in aid after Gavi graduation.

**HOW THIS STUDY MIGHT AFFECT RESEARCH, PRACTICE OR POLICY**

- Our study observed that some countries may be at risk of losing donor resources for health from a multitude of sources around the same time. However, countries that have a strategic interest in bilateral donors may be spared from such funding cliffs. This research has important implications for global health donors’ funding policies and approaches in addition to recipient countries’ transition planning.

## Introduction

Middle-income status is a threshold often used by foreign aid donors to measure a country’s ability to finance its own government or health systems. Crossing this threshold can act as a trigger to reduce or withdraw foreign aid, known as transition or graduation from official development assistance (ODA). Despite aggregate economic gains, the World Bank^1^ finds that middle-income countries are still home to most of the world’s poorest people. Additionally, these types of aggregate income-based thresholds can result in donors behaving similarly to each other, ultimately leading to simultaneous ‘transitions’ out of country support. Even when a country is considered to have reached middle-income status, and potentially perceived as capable of ‘self-reliance’, Silverman^2^ and Yamey *et al*^3^ find that the simultaneous loss of external funding from a variety of sources can make it difficult to fill financial gaps with domestic resources.

McDade *et al*^4^ find that multilateral donors tend to have more explicit graduation policies than bilateral donors.^4^ Gavi, the Vaccine Alliance (Gavi), has a clear graduation approach and policy, informed by need-driven indicators such as gross domestic product (GDP) per capita and vaccine coverage rates, a paper by Bharali^5^ finds. Although not the only factor involved in decision-making, the ‘middle-income threshold’ is a major determinant in Gavi’s approach. Gavi’s support is designed to primarily target lower-income countries,^6^ and once a country crosses over the low-income eligibility threshold, which is based on average gross national income per capita, countries begin the accelerated transition phase and phase out of support. If bilateral donors are using the same set of criteria or following Gavi’s lead, countries may see their aid decrease from multiple donors once they meet Gavi graduation criteria.

Bilateral donors may not apply graduation criteria equally. Recent scholarship by Bermeo^7^ shows that bilateral donors behave strategically. They may be more likely to continue their funding, or even fill a funding gap left by Gavi graduation, if they have a strategic interest in the recipient. This continued support by bilateral donors of strategically important countries would result in less strategic countries being disproportionately disadvantaged by graduation from eligibility in multilateral programmes.

Gulrajani and Silcock^8^ of The Overseas Development Institute (ODI) highlight this phenomenon in the 2020 ‘Principled Aid Index’, which ranks donors on how they strike a balance between global solidarity and protecting their national interests. ODI uses the framing ‘principled nationalism’ to describe when foreign aid protects donor interests while advancing global values. This phenomenon has yet to be explored specifically within the health sector.

We hypothesise that bilateral donors will follow Gavi’s lead, except in recipient countries where donors have significant strategic interests. We anticipate that these countries are likely to continue receiving bilateral health aid even after a transition from Gavi, and will not be left behind. Countries that are not strategically important to bilateral donors are unlikely to continue receiving significant bilateral health aid after Gavi graduation and could be at risk of a fiscal cliff from a multitude of donors. Here, this study aims to address three research questions: (1) Have bilateral donors followed Gavi’s decision in financing countries (and in transitioning out of countries)? (2) Have bilateral donors treated different graduated countries differently, based on ‘strategic interest’? and (3) What kinds of countries were more vulnerable to ‘simultaneous’ transitions and financial cliffs after Gavi transition?

## Methods

This is an observational dyadic analysis using longitudinal data. To assess the impact of Gavi graduation and funding on bilateral donors’ behaviour, we collected country-level ODA and health ODA data for all Gavi-eligible countries between 2009 and 2018, and paired donor and recipient country in a specific year to conduct dyadic analysis. We selected Gavi as our multilateral donor because it is a health-focused multilateral donor, has an explicit graduation policy and has a large enough sample of countries that have graduated. Our models incorporate health-sector specific variables into the general aid allocation framework used by Bermeo.^9 10^

### Sample and data

Our main analysis examines dyadic aid disbursement between 2009 and 2017. Our unit of analysis is an aid disbursement from a donor to a recipient for a given year; for example, funding from the USA to Kenya for health for the year 2009. We began our analysis in 2009 because Gavi started reporting to the Organization for Economic Cooperation and Development (OECD) in 2009, and the latest year of data available for most variables is 2018. This ending time point also avoids confounding factors created by the COVID-19 pandemic that might not be indicative of broader patterns. However, many dependent variables have been lagged 1 year to allow policy effect on aid disbursement (ie, GDP in 2017 will affect the aid disbursement in 2018.) We analyse country-level data for 17 OECD bilateral donors to 77 Gavi eligible countries.

We select bilateral donors that contribute at least 1% of total bilateral ODA or bilateral health ODA during the study period. For recipient countries, we analyse countries that either currently are, or were, eligible for Gavi funding during the study period. The list of donors and countries can be found in online supplemental appendices 1 and 2. A full list of variables and sources can be found in table 1.

10.1136/bmjgh-2023-012953.supp1 Supplementary data

10.1136/bmjgh-2023-012953.supp2 Supplementary data

**Table 1:** List of variables

	Definition	Approach	Data source
*Dependent variables*			
All sector ODA	Purpose code: total all sectors (1000) (Million constant 2017 US$)	Logged (1+)	OECD Creditor Reporting System (CRS) database^21^
Health ODA	Purpose codes: sum of health (120), populational policies and programmes (130) and social mitigation of HIV/AIDS (16 064) (Million constant 2017 US$)	Logged (1+)	
*Explanatory variables*			
Gavi graduate	Years of and before the graduation were coded as ‘0’ and each subsequent year after graduation were coded as ‘1’ for a given recipient (see online supplemental appendix 1 for full list)	Lagged	Gavi website^22^
Gavi disbursements	Gavi ODA disbursements, recipient country level (Million constant 2017 US$)	Logged (1+) and lagged	OECD CRS database^21^
*Recipient–donor relationship control variables*			
Distance	Distance between donor and recipient capital cities (km)	Logged	EUGene v. 3.2 software^23^
Donor imports	Donor imports from the recipient in a given year (Million constant 2017 US$)	Logged and lagged	International Monetary Fund’s Direction of Trade Statistics^24^
Migrants	The population stock of migrants from a recipient country residing in a donor country in a given year	Logged (1+) and lagged	OECD^25^
Colony	1 if the recipient was ever a colony of the donor and 0 otherwise		Coded from the Central Intelligence Agency World Factbook^26^
US military	Military assistance a country receives from the USA in a given year (Million constant 2017 US$)	Logged (1+)	US Agency for International Development Greenbook^27^
Donor exports	Donor exports to a recipient in a given year (Million constant 2017 US$)	Logged and lagged	International Monetary Fund’s Direction of Trade Statistics^24^
*Recipient control variables*			
Population	Population size of recipient country (Million)	Logged (1+)	Penn World Table version 6.3^28^
Income	Gross domestic product per capita (Constant 2017 US$)	Logged and lagged	Penn World Table version 6.3^28^
Disaster	The total number of people affected or killed as a result of natural disasters in a country in a given year	Logged	Center for Research on the Epidemiology of Disasters (CRED) International Disaster Database^29^
Civil war	1 if a recipient experienced one or more civil wars in a given year and 0 otherwise	Lagged	The Peace Research Institute Oslo (PRIO) Armed Conflict Dataset^30^
Democracy	Average of a recipient’s score on the civil liberties and political rights; 1 is most democratic and 7 least democratic	Lagged	The Freedom House’s Freedom in the World data set^31^
U5MR	Mortality rate, under-5 (per 1000 live births)	Lagged	World Development Indicators^32^
DTP3	Diphtheria-tetanus-pertussis (DTP3) immunisation coverage among 1 year-olds (%)	Lagged	WHO^33^
HDI	Human Development Index (%)	Lagged	United Nations Development Programme^34^

Notes: All financial data is reported in constant 2017 US$, using conversion methods as outlined in Turner *et al*.^35^ We used the natural log of (one plus) to adjust the skewed distribution of variables. Some variables have been lagged 1 year to allow policy effect. Gavi graduate and Gavi disbursement have been lagged 1, 3 and 5 years, respectively, to observe the effect in the short and longer term.

ODA, official development assistance; OECD, Organization for Economic Cooperation and Development.

We checked for patterns in how bilateral aid is channelled through multilateral agencies (‘bi/multi’ channelling) to ensure that we were not inadvertently double counting or misinterpreting our findings if, for example, donors continued to fund Gavi graduates through bi/multi channels. Although bi/multi channels are used by some health donors, Gavi does not seem to be a channel that is commonly used. There are some projects that report bi/multi aid where Gavi is the multilateral channel, although these are so infrequent that we believe these may be reported erroneously to the OECD system.

#### Dependent variables

To explore donor behaviour within the health sector and beyond, we assess total ODA across all sectors (OECD purpose code 1000) and health sector ODA (OECD purpose codes 120 and 130). Our goal in assessing both types of ODA is to observe if Gavi funding affects total ODA and health sector ODA differently. We recognise the possibility of aid fungibility, where resources designated for one sector may be used in another. Assessing both types of ODA helps us minimise the potential for missing such a phenomenon within our data. We report country-level bilateral disbursements since these are representative of funds received; a comparable analysis with commitments shows no substantive difference in findings.

#### Key explanatory variables

We used Gavi graduation status, which is a binary variable, as the first explanatory variable to observe the impact of Gavi funding level on other bilateral donors. Years of and before the graduation were coded as ‘0’ and each subsequent year after graduation was coded as ‘1’ for a given recipient. There are 23 countries that have graduated from Gavi’s support (online supplemental appendix 1) and the year of graduation has been used to set up the Gavi graduation status.

We include Gavi disbursement, which is a continuous variable, as another explanatory variable to assess the impact of Gavi’s support on other bilateral donors’ behaviour.

#### Control variables

We use a number of control variables from Bermeo.^10^ In particular, we use several donor–recipient relationship variables that represent potential strategic relationships between donor and recipient. These variables include distance between donor and recipient country, migration from recipient to donor, donor imports, US military assistance, donor exports and status as a former colony of the donor (table 1). We also include variables to capture recipient characteristics, including population, income level, natural disasters, civil war and democracy.

Given our focus on the health sector, we have added several health-specific variables. We use indicators that Gavi frequently uses in its graduation policy, such as diphtheria-tetanus-pertussis (DTP3) coverage, in addition to commonly used health indicators like the under-five mortality rate (U5MR) and the Human Development Index (HDI). In selecting control variables, efforts were made to include measures that are available for most country years.

Gavi graduation status is interacted with donor–recipient relationship variables to assess the impact of these bilateral relationships on a recipient’s vulnerability to losing bilateral aid when it graduates from Gavi eligibility.

### Data analysis and robustness check

Following the guidance outlined in Wooldridge,^11^ we use the Ordinary Least Square (OLS) regression model on all sector ODA and Tobit models on health ODA with left censoring at 0, fixed effects on donors and years, and robust standard errors clustered on dyad.^10^ OLS regression model was selected for all sector ODA which is a continuous variable. We chose the Tobit model on health ODA because it is a limited dependent variable that is continuous over most of its distribution but has approximately one-third valued 0.^12^ The Tobit model assumes a single decision where ‘0’ is treated as a corner solution and the Tobit model is estimated by maximum likelihood. There is no consensus in aid literature about the model selection. Tobit models were presented in the main text for their simplicity in interpretation. Fixed effects for year and donor are included in all Tobit and OLS models, following the rationale outlined by Bermeo.^10^ In short, this analysis assumed that donors’ behaviours are non-random and follow fixed patterns across different years.

We also use alternative models as robustness check, including Tobit model with robust standard errors clustered on donors, and two-part model, when applicable. Two-part model is another approach to handle data with massive 0s. Two-part model is a selection equation model using probit or logit with a binary dependent variable indicating whether or not the observation has positive values for aid, followed by a level equation estimated using OLS and restricted to those observations with strictly positive aid values. The results from two-part models were similar to the Tobit model thus we presented the two-part model in the online supplemental appendix.

To identify features that might make recipient countries more vulnerable to funding cliffs, we examine the coefficients on interaction terms between Gavi graduation status and donor–recipient relationship variables. When focusing on individual donors, we examine the top three bilateral health ODA donors (USA, UK and Japan). We also conducted analysis on the only bilateral donor that does not contribute to Gavi (Belgium) among the 17 donors included in this study.

We use Stata SE 15.0 for all analyses.

### IRB

This study used publicly available data without any individual information. Ethical clearance is not applicable.

## Results

We find that Gavi graduation has a negative association with bilateral all sector ODA (p<0.05) (table 2, M1) and health ODA (p<0.05) (table 2, M3). This means that after a country graduates from Gavi, its ODA from bilateral donors declines, on average. Assuming all other factors remain the same, a country would receive $3.1 million less all sector ODA on average from a bilateral donor, and $0.6 million less health ODA, after they graduate from Gavi than prior to graduation. Graduation tended to have a larger effect in percentage term on health ODA than all sector ODA on average.

**Table 2:** Impact of Gavi funding and graduation on bilateral donor ODA

Variable	All sector ODA		Health ODA	
	(M1)	(M2)	(M3)	(M4)
Gavi graduate	−0.371***		−0.471***	
	(0.107)		(0.115)	
Gavi disbursement		0.138***		0.164***
		(0.0292)		(0.0304)
**Recipient-donor relationship variables**				
Distance	−0.588***	−0.571***	−0.444***	−0.426***
	(0.102)	(0.0999)	(0.0962)	(0.0941)
Donor imports	−0.0186	−0.0182	0.0199	0.0195
	(0.0233)	(0.0232)	(0.0240)	(0.0239)
Migrants	0.0340	0.0298	0.0437*	0.0380
	(0.0234)	(0.0236)	(0.0242)	(0.0240)
Colony	0.244***	0.243***	0.0961	0.0971
	(0.0917)	(0.0916)	(0.0932)	(0.0929)
US military	0.116***	0.101***	0.0321	0.0150
	(0.0222)	(0.0220)	(0.0220)	(0.0218)
Donor exports	−0.0253	−0.0181	−0.0503*	−0.0412
	(0.0264)	(0.0261)	(0.0264)	(0.0261)
**Recipient variables**				
Population	0.420***	0.357***	0.375***	0.298***
	(0.0349)	(0.0379)	(0.0347)	(0.0362)
GDP per capita	−0.380***	−0.395***	−0.485***	−0.502***
	(0.0779)	(0.0774)	(0.0775)	(0.0769)
Disaster	0.0236***	0.0215***	0.00754*	0.00541
	(0.00426)	(0.00427)	(0.00423)	(0.00425)
Civil war	0.139*	0.116	−0.0383	−0.0687
	(0.0730)	(0.0718)	(0.0723)	(0.0717)
Democracy	−0.0499**	−0.0442**	−0.0739***	−0.0667***
	(0.0206)	(0.0204)	(0.0197)	(0.0193)
U5MR	−0.00490***	−0.00464***	−0.00204	−0.00161
	(0.00170)	(0.00169)	(0.00167)	(0.00165)
DTP3 coverage	0.000857	0.000108	0.00642***	0.00557**
	(0.00233)	(0.00233)	(0.00235)	(0.00235)
HDI	−0.662	−0.0302	−1.079*	−0.297
	(0.586)	(0.573)	(0.584)	(0.573)
Constant	9.317***	9.013***	7.120***	6.499***
	(1.194)	(1.187)	(1.311)	(1.297)
Observations	10 275	10 247	10 245	10 247
R-squared	0.462	0.464		
Model	OLS	OLS	Tobit	Tobit
Fix effects (FE)	Donor, year	Donor, year	Donor, year	Donor, year
Cluster	Dyad	Dyad	Dyad	Dyad

Notes: Dependent variable is the log of (one plus) all sector aid disbursement (or health aid disbursement) from donor to recipient in year t. All variables except Gavi Graduate, Colony, Civil War, Democracy, U5MR, DTP3 coverage and HDI are measured in natural logs. *p<0.10, **p<0.05, ***p<0.01.

Two-part model for health ODA can be found in online supplemental appendix 4 Table S4.1.

DTP3, diphtheria-tetanus-pertussis; GDP, gross domestic product; HDI, Human Development Index; ODA, official development assistance; U5MR, under-five mortality rate.

10.1136/bmjgh-2023-012953.supp4 Supplementary data

Additionally, we find that Gavi disbursement was positively associated with bilateral ODA (p<0.05) (table 2, M2) and health ODA (p<0.05) (table 2, M4). Assuming all other factors remain the same, for every additional 1% a country would receive from Gavi, it would receive 0.14% more ODA and 0.16% more health ODA from individual bilateral donors. Again, Gavi’s disbursement brought more percentage of change to health ODA than to total ODA.

Two-part models for health ODA have similar findings to the main models in table 2 that Gavi graduates receive less bilateral health ODA and may be less likely to receive bilateral health ODA (online supplemental appendix 4 Table S4.1, M3-1 and M3-2). Higher amounts of Gavi disbursement are associated with a greater likelihood of receiving bilateral health ODA, and with receiving more bilateral health ODA conditional on receiving Gavi disbursement (online supplemental appendix 4 table S4.1, M4-1 and M4-2).

Several variables indicating the recipient’s strategic interest to the donor are also associated with total bilateral ODA (table 2 M1) and health ODA (table 2 M3 and M4). We found a positive association between bilateral aid (all sector ODA and health ODA) and sending migrants to donor countries, being a former colony, and receiving US military support, though not all of the associations reached statistical significance. Distance from a donor (p<0.05) and the level of exports received from a donor are negatively associated with bilateral all sector ODA and health ODA, indicating bilateral donors tend to provide more ODA to nearby countries and countries receiving fewer exports from the donor.

Several specific features of Gavi countries are associated with more total bilateral ODA and health ODA (table 2). We see a positive association with population (p<0.05), disaster and DTP3 coverage, and a negative association with GDP per capita (p<0.05), democracy (p<0.05), under-five mortality and HDI. This means that countries with large populations, that have experienced natural disasters, and have higher DTP3 coverage receive more all sector and health bilateral ODA. Additionally, countries that are more democratic, have lower GDP per capita, lower under-five mortality rates and lower HDI receive more all sector and health bilateral ODA. This signals that higher values of some measures of recipient need are associated with more bilateral ODA and health ODA.

We compared the impact of Gavi transition on bilateral ODA from USA, UK, Japan and Belgium, respectively (online supplemental appendix 3). For a country that graduated from Gavi, it tended to have less all sector ODA from USA and Japan (p<0.05) but more from UK and Belgium than it did before graduation (online supplemental appendix 3 table S3.1). Controlling for other variables, country would receive less health ODA from USA (p<0.05), UK and Japan after their Gavi graduation, except for Belgium (online supplemental appendix 3 table S3.2). Gavi disbursement was positively associated with all sector and health ODA from the four donors (p<0.05 for USA, UK and Japan) (online supplemental appendix 3). There were several clear areas of homogeneity and heterogeneity across variables (online supplemental appendix 3). Positive associations with all types of ODA were found for migrants (except for Belgium), US military, population, civil war (except for Belgium) and DTP3 (except for USA), while negative associations were observed for distance (except for UK and Belgium), GDP per capita and democracy. Former colony status had negative association with total ODA and health ODA in USA and UK but positive for Japan and Belgium. US military assistance was positively associated with all sector and health ODA from USA and Japan but the association was negative with UK and Belgium’s health aid (online supplemental appendix 3).

10.1136/bmjgh-2023-012953.supp3 Supplementary data

In the longer term, Gavi graduate remains a negative association with bilateral all sector ODA (p<0.05) and health ODA (p<0.05) (table 3) and 5 years’ impact is larger than 3 year’s impact. Gavi disbursement also remain positive association with bilateral ODAs (p<0.05) (table 3) and the effect size tends to be smaller in longer terms.

**Table 3:** Time effect of Gavi funding and graduation on bilateral donor ODA

Variable	All sector ODA				Health ODA			
	Lag 3 years	Lag 3 years	Lag 5 years	Lag 5 years	Lag 3 years	Lag 3 years	Lag 5 years	Lag 5 years
	M5	M6	M7	M8	M9	M10	M11	M12
Gavi graduate	−0.551***		−0.687***		−0.619***		−0.621***	
	(0.156)		(0.186)		(0.170)		(0.200)	
Gavi disbursement		0.139***		0.126***		0.155***		0.141***
		(0.0303)		(0.0333)		(0.0319)		(0.0366)
Observations	7969	7942	5877	5852	7969	7942	5877	5877
R-squared	0.472	0.474	0.489	0.491				
Model	OLS	OLS	OLS	OLS	Tobit	Tobit	Tobit	Tobit
Fix effects (FE)	Donor, year	Donor, year	Donor, year	Donor, year	Donor, year	Donor, year	Donor, year	Donor, year
Cluster	Dyad	Dyad	Dyad	Dyad	Dyad	Dyad	Dyad	Dyad

Notes: Dependent variable is the log of (one plus) all sector aid disbursement (or health aid disbursement) from donor to recipient in year t. *p<0.10, **p<0.05, ***p<0.01. Other variables are included in the model and can be found in online supplemental appendix 4 table S4.2.

ODA, official development assistance.

Finally, we interacted Gavi graduation with selected variables to further investigate the joint influence of Gavi graduation and the aforementioned factors on bilateral donor behaviour (table 4). We found that former colonies do not see a decline in aid after Gavi graduation.

**Table 4:** Impact of Gavi transition on different types of bilateral ODA and country vulnerability

Variable	All sector ODA	Health ODA	All sector ODA	Health ODA	All sector ODA	Health ODA	All sector ODA	Health ODA	All sector ODA	Health ODA	All sector ODA	Health ODA	All sector ODA	Health ODA
	M13	M14	M15	M16	M17	M18	M19	M20	M21	M22	M23	M24	M25	M26
Gavi graduate	−0.563	−0.525	−1.262	−1.227	−0.227	−0.347	−0.217	0.0278	−0.457***	−0.511***	−0.246	−0.423**	−0.158	−0.299
	(1.715)	(1.730)	(1.204)	(1.288)	(0.180)	(0.218)	(0.624)	(0.624)	(0.135)	(0.146)	(0.163)	(0.179)	(0.192)	(0.236)
**Interactions**														
Gavi graduate * distance	0.0502	0.0778	0.105	0.0889										
	(0.188)	(0.180)	(0.141)	(0.150)										
Gavi graduate* donor imports	0.152	0.159			−0.0210	−0.0170								
	(0.106)	(0.130)			(0.0290)	(0.0304)								
Gavi graduate* migrants	0.00164	−0.0369					−0.0127	−0.0403						
	(0.0527)	(0.0589)					(0.0538)	(0.0533)						
Gavi graduate* colony	0.255	0.0552							0.358**	0.171				
	(0.217)	(0.250)							(0.179)	(0.208)				
Gavi graduate * US military	−0.0103	0.0292									−0.0710	−0.0267		
	(0.0696)	(0.0693)									(0.0646)	(0.0667)		
Gavi graduate * donor exports	−0.196*	−0.192											−0.0312	−0.0240
	(0.113)	(0.145)											(0.0312)	(0.0334)
Observations	10 275	10 275	10 275	10 275	10 275	10 275	10 275	10 275	10 275	10 275	10 275	10 275	10 275	10 275
R-squared	0.463		0.462		0.462		0.462		0.462		0.462		0.462	
Model	OLS	Tobit	OLS	Tobit	OLS	Tobit	OLS	Tobit	OLS	Tobit	OLS	Tobit	OLS	Tobit
FE	Donor, year	Donor, year	Donor, year	Donor, year	Donor, year	Donor, year	Donor, year	Donor, year	Donor, year	Donor, year	Donor, year	Donor, year	Donor, year	Donor, year
Cluster	Dyad	Dyad	Dyad	Dyad	Dyad	Dyad	Dyad	Dyad	Dyad	Dyad	Dyad	Dyad	Dyad	Dyad

Notes: dependent variable is the log of (one plus) all sector aid disbursement (or health aid disbursement) from donor to recipient in year t. *p<0.10, **p<0.05, ***p<0.01. Other variables are included in the model and can be found in online supplemental appendix 4 table S4.3.

FE, fixed effects; ODA, official development assistance.

## Discussion

Our study found evidence supporting our hypothesis that bilateral donors generally follow Gavi’s lead. Countries that receive more ODA from Gavi also receive more bilateral ODA and Gavi graduate countries tend to see declines in bilateral health and all sector ODA. However, this trend does not occur for countries that are of strategic interest to bilateral donors. This finding is similar to previous work that donors have strategic interests when allocating their ODA.^10 13^ In particular, a country that is a former colony is likely to see sustained funding despite Gavi graduation status. Overall, need-based recipient indicators matter for non-graduates: these factors correspond to more ODA. This is also aligned with another work.^14^ Yet former colonial status matters for sustained funding on graduation.

This research has several implications. First, it shows that donors tend to behave similarly. It is therefore plausible that simultaneous transitions, such as those highlighted by Silverman,^2^ could leave many countries in a vulnerable financing position. Such transitions and reduction of ODA also have a large impact in the longer terms. Planning and preparing for transitions out of aid should be given more attention, particularly for those donors who do not have explicit or transparent approaches. In the next decade, over a dozen of lower-middle income countries are expected to graduate from donor assistance, including the most populous countries in Asia (India) and Africa (Nigeria).^3^ As our findings indicated, those countries are suspected to experience fund cliff after simultaneous graduations from multiple donors. Without properly managing the transition process, backsliding effects might occur, leaving a large number of households without proper health services.^15^ Several plans might support the transition process including developing a transition plan in advance, shifting ODA on vertical programmes towards developing a more sustaining and resilient health system and mobilising domestic resources to health sector.^16 17^

Second, pinning ODA allocation to aggregate or national-level indicators may lead to funding declines in some countries despite large ‘pockets of poverty’. Most of the world’s poor live in middle-income countries, many of which have lost ODA despite continuing to have large pockets of high mortality in these regions of poverty. There is an opportunity to rethink how health ODA should be allocated to best serve the poor, in whichever country they may be. For example, Gavi and IDA have specifically incorporated subnational poverty indicators or broader subnational poverty focus into their aid policy and routine monitoring process and such subnational and poverty-oriented approaches should be considered by more donors to tackle the poverty issue in middle-income countries.^18^ Particular focus should be paid to transitioning countries that may not be of strategic interest to bilateral donors, as these settings will be the most at risk of funding cliffs.

The Commission on Investing in Health^19^ has also argued that one important way for donors to help improve the health of the poor living in middle-income countries that have graduated from ODA is to fund global public goods for health. These goods include research and development for diseases of poverty, polio and malaria elimination, pooled procurement of drugs and vaccines to bring down drug prices. Schäferhoff *et al*^20^ argue that ‘countries such as China and India would substantially benefit from collective purchasing of commodities, market shaping to reduce drug prices and increased international efforts to control multidrug-resistant tuberculosis’.

While we aimed to select the most appropriate methods to perform our analysis, we are aware of several limitations in our work. First, we do not analyse the role of emerging bilateral donors, such as China, given data availability issues. Second, some aid data were not included in the model due to missing values. However, missing values accounted for less than 1% of the total sample size, and therefore we found it appropriate to exclude. Finally, while we aim to include variables and proxies that are both used in previous similar analyses and appropriate for our research question, our models may not have captured some important factors. However, our R-square indicates that our models perform well.

## Conclusion

Bilateral donors behave in a similar manner to Gavi when it comes to funding the health sector in low and middle-income countries. Therefore, some countries may be at risk of losing donor resources for health from a multitude of sources around the same time. However, countries that are of strategic interest to bilateral donors may be spared from such funding cliffs. This research has important implications for global health donors’ funding policies and approaches in addition to recipient countries’ transition planning.

## Data Availability

Data are available in public, open access repositories.
