# Supplementary material for: Strategic donor behaviour and country vulnerability in health aid transitions
Source: BMJ Glob Health. 2023 Nov 8;8(11):e012953. doi: 10.1136/bmjgh-2023-012953 (PMC10632813; doi:10.1136/bmjgh-2023-012953)
Supplement: Supplementary data [file bmjgh-2023-012953supp001.pdf]

Appendix 1 Gavi eligible countries included in analysis

77 countries are/were eligible to receive Gavi support during the study period and among the 77 countries, 23 countries have graduated from Gavi support.

	Gavi eligible countries	Graduation year	Samples included in analysis (and reasons for exclusion)
1	Afghanistan		170
2	Albania	2013 *	150
3	Angola	2017	157
4	Armenia	2017	148
5	Azerbaijan	2017	126
6	Bangladesh		170
7	Benin		149
8	Bhutan	2015	114
9	Bolivia	2017	166
10	Bosnia and Herzegovina	2011*	164
11	Burkina Faso		163
12	Burundi		163
13	Cambodia		166
14	Cameroon		152
15	Central African Republic		149
16	Chad		141
17	China (People's Republic of)	2006*	168
18	Comoros		64
19	Congo		128
20	Côte d'Ivoire		149
21	Cuba	2016	68
22	Democratic People's Republic of Korea		Excluded: HDI and GDP data not available
23	Democratic Republic of the Congo		Excluded: Distance not available
24	Djibouti		62 (2012-2017): GDP data incomplete
25	Eritrea		73 (2009-2012): GDP data incomplete
26	Ethiopia		170
27	Gambia		138
28	Georgia	2017	151
29	Ghana		170
30	Guinea		138
31	Guinea-Bissau		129
32	Guyana	2016	83
33	Haiti		154
34	Honduras	2015	161
35	India		153
36	Indonesia	2016	165
37	Kenya		170
38	Kiribati	2016	46

39	Kyrgyzstan		128
40	Lao People's Democratic Republic		139
41	Lesotho		124
42	Liberia		153
43	Madagascar		143
44	Malawi		161
45	Mali		160
46	Mauritania		131
47	Moldova	2016	141
48	Mongolia	2015	143
49	Mozambique		170
50	Myanmar		165
51	Nepal		170
52	Nicaragua	2020	163
53	Niger		158
54	Nigeria		168
55	Pakistan		170
56	Papua New Guinea		91
57	Rwanda		165
58	Sao Tome and Principe		68
59	Senegal		160
60	Sierra Leone		160
61	Solomon Islands		73
62	Somalia		Excluded: HDI not available
63	South Sudan		Excluded: Distance not available
64	Sri Lanka	2015	163
65	Sudan		170
66	Tajikistan		139
67	Tanzania		168
68	Timor-Leste	2017	Excluded: Distance not available
69	Togo		141
70	Turkmenistan	2007*	72
71	Uganda		170
72	Ukraine	2010*	158
73	Uzbekistan	2020	113
74	Viet Nam	2019	167
75	Yemen		158
76	Zambia		166
77	Zimbabwe		170

Source: Gavi website. Gavi graduation year indicates the final year of support before becoming fully self-financing.
**Denotes countries where support ended prior to the establishment of a formal transition policy. These countries transitioned when their GNI per capita exceeded the eligibility threshold.*
The following eligible countries are completely or partially excluded from the analysis due to missing values: Timor-Leste, South Sudan, DRC, Somalia, Eritrea (ends in 2012), DPRK
