# Supplementary material for: Strategic donor behaviour and country vulnerability in health aid transitions
Source: BMJ Glob Health. 2023 Nov 8;8(11):e012953. doi: 10.1136/bmjgh-2023-012953 (PMC10632813; doi:10.1136/bmjgh-2023-012953)
Supplement: Supplementary data [file bmjgh-2023-012953supp002.pdf]

Appendix 2 Bilateral donors included in analysis (n=17)

	**Bilateral donors**
1	Australia
2	Belgium
3	Canada
4	Denmark
5	France
6	Germany
7	Ireland
8	Italy
9	Japan
10	Korea
11	Netherlands
12	Norway
13	Spain
14	Sweden
15	Switzerland
16	United Kingdom
17	United States

Mao W, *et al. BMJ Glob Health* 2023; 8:e012953. doi: 10.1136/bmjgh-2023-012953
