# Supplementary material for: Strategic donor behaviour and country vulnerability in health aid transitions
Source: BMJ Glob Health. 2023 Nov 8;8(11):e012953. doi: 10.1136/bmjgh-2023-012953 (PMC10632813; doi:10.1136/bmjgh-2023-012953)
Supplement: Supplementary data [file bmjgh-2023-012953supp004.pdf]

Appendix 4 Alternative models

Table S4.1 Two-part analysis on health ODA

Variables	M3-1	M3-2	M4-1	M4-2
Gavi graduate	-0.170	-0.359***		
	(0.107)	(0.120)		
Gavi disbursement			0.0923***	0.116***
			(0.0283)	(0.0291)
**Recipient-donor relationship variables**				
Distance	-0.0710	-0.288***	-0.0755	-0.300***
	(0.0651)	(0.0806)	(0.0656)	(0.0822)
Donor imports	0.00308	0.00835	0.00485	0.00872
	(0.0241)	(0.0219)	(0.0241)	(0.0218)
Migrants	0.0455**	0.0199	0.0486**	0.0238
	(0.0224)	(0.0226)	(0.0226)	(0.0225)
Colony	0.0768	0.0108	0.0863	0.00652
	(0.0787)	(0.0825)	(0.0787)	(0.0824)
US military	-0.0102	0.0252	0.000675	0.0366*
	(0.0194)	(0.0201)	(0.0196)	(0.0201)
Donor exports	-0.00889	-0.0426*	-0.0149	-0.0488**
	(0.0264)	(0.0237)	(0.0265)	(0.0239)
**Recipient variables**				
Population	0.163***	0.213***	0.203***	0.268***
	(0.0351)	(0.0324)	(0.0334)	(0.0307)
GDP per capita	-0.340***	-0.287***	-0.338***	-0.275***
	(0.0740)	(0.0797)	(0.0738)	(0.0791)
Disaster	0.00276	0.00468	0.00373	0.00610
	(0.00416)	(0.00398)	(0.00414)	(0.00396)
Civil war	-0.0117	-0.0636	0.0103	-0.0401
	(0.0686)	(0.0658)	(0.0687)	(0.0664)
Democracy	-0.0452**	-0.0466***	-0.0501***	-0.0511***
	(0.0191)	(0.0173)	(0.0192)	(0.0174)
U5MR	6.27e-05	-0.00194	9.91e-06	-0.00227
	(0.00152)	(0.00146)	(0.00152)	(0.00148)
DTP3 coverage	0.00533**	0.00118	0.00592**	0.00179
	(0.00233)	(0.00219)	(0.00233)	(0.00218)
HDI	0.0688	-0.518	-0.312	-1.076*
	(0.544)	(0.634)	(0.531)	(0.639)
Year	-0.0262***		-0.0123**	
	(0.00667)		(0.00613)	
Constant	55.11***	5.957***	27.44**	6.263***
	(13.51)	(1.029)	(12.49)	(1.025)
Observations	10,275	6,926	10,247	6,906
R-squared		0.396		0.399
Model	Probit	OLS	Probit	OLS
Fix Effects (FE)		Donor, Year		Donor, Year
Cluster	Dyad	Dyad	Dyad	Dyad

Notes: Dependent variable is the log of (one plus) health aid disbursement from donor to recipient in year t. All variables except Gavi Graduate, Colony, Civil War, Democracy, U5MR, DTP3 coverage and HDI are measured in natural logs. *p<0.10, **p<0.05, ***p<0.01.

Table S4.2 Full models of time effect of Gavi funding and graduation on bilateral donor ODA

Variable	All sector ODA				Health ODA			
	Lag 3 years M6	Lag 3 years M5	Lag 5 years M8	Lag 5 years M7	Lag 3 years M10	Lag 3 years M9	Lag 5 years M12	Lag 5 years M11
Gavi graduate	-0.551***		-0.687***		-0.619***		-0.621***	
	(0.156)		(0.186)		(0.170)		(0.200)	
Gavi disbursement		0.139***		0.126***		0.155***		0.141***
		(0.0303)		(0.0333)		(0.0319)		(0.0366)
**Recipient-donor relationship variables**								
Distance	-0.608***	-0.581***	-0.636***	-0.616***	-0.450***	-0.424***	-0.440***	-0.427***
	(0.107)	(0.105)	(0.108)	(0.107)	(0.101)	(0.0978)	(0.102)	(0.100)
Donor imports	-0.0563**	-0.0486*	-0.0822***	-0.0753***	-0.0052	0.0021	-0.0109	-0.0088
	(0.0250)	(0.0250)	(0.0275)	(0.0274)	(0.0264)	(0.0263)	(0.0301)	(0.0300)
Migrants	0.0207	0.0211	-0.0080	-0.0031	0.0337	0.0330	0.0345	0.0347
	(0.0255)	(0.0258)	(0.0269)	(0.0271)	(0.0263)	(0.0262)	(0.0281)	(0.0279)
Colony	0.225**	0.245**	0.237**	0.270***	0.0928	0.111	0.0968	0.123
	(0.0959)	(0.0956)	(0.0998)	(0.0999)	(0.0990)	(0.0980)	(0.104)	(0.103)
US military	0.105***	0.0923***	0.0929***	0.0835***	0.0264	0.0124	0.0265	0.0126
	(0.0232)	(0.0226)	(0.0242)	(0.0237)	(0.0235)	(0.0231)	(0.0244)	(0.0242)
Donor exports	0.0098	0.0095	0.0238	0.0228	-0.0271	-0.0268	-0.0259	-0.0219
	(0.0281)	(0.0276)	(0.0299)	(0.0296)	(0.0287)	(0.0281)	(0.0307)	(0.0303)
**Recipient variables**								
Population	0.446***	0.372***	0.491***	0.419***	0.391***	0.307***	0.406***	0.334***
	(0.0373)	(0.0397)	(0.0395)	(0.0412)	(0.0367)	(0.0380)	(0.0390)	(0.0396)
GDP per capita	-0.311***	-0.349***	-0.176	-0.218**	-0.443***	-0.480***	-0.411***	-0.443***
	(0.0909)	(0.0913)	(0.111)	(0.111)	(0.0899)	(0.0893)	(0.115)	(0.114)
Disaster	0.0237***	0.0234***	0.0170***	0.0188***	0.0053	0.0051	-0.0015	-0.0002
	(0.0046)	(0.0046)	(0.0049)	(0.0048)	(0.0047)	(0.0047)	(0.0051)	(0.0051)
Civil war	0.100	0.0983	0.0882	0.102	-0.0428	-0.0511	-0.0656	-0.0715
	(0.0787)	(0.0773)	(0.0832)	(0.0827)	(0.0781)	(0.0780)	(0.0835)	(0.0850)
Democracy	-0.0426**	-0.0456**	-0.0315	-0.0408*	-0.0676***	-0.0698***	-0.0642***	-0.0701***
	(0.0214)	(0.0211)	(0.0220)	(0.0218)	(0.0206)	(0.0202)	(0.0214)	(0.0211)
U5MR	-0.0057***	-0.0053***	-0.0101***	-0.0096***	-0.0030	-0.0025	-0.0043*	-0.0039
	(0.0019)	(0.0019)	(0.0024)	(0.0024)	(0.0018)	(0.0018)	(0.0024)	(0.0024)
DTP3 coverage	-0.0002	-0.0010	-0.0031	-0.0031	0.0057**	0.0048*	0.0048*	0.0041
	(0.0024)	(0.0024)	(0.0025)	(0.0025)	(0.0025)	(0.0025)	(0.0026)	(0.0026)
HDI	-1.152	-0.415	-2.717**	-2.091*	-1.601**	-0.764	-2.258**	-1.565
	(0.785)	(0.783)	(1.083)	(1.094)	(0.753)	(0.750)	(1.095)	(1.099)
Constant	9.490***	9.194***	9.785***	9.658***	7.338***	6.802***	7.497***	7.127***
	(1.221)	(1.212)	(1.251)	(1.259)	(1.352)	(1.329)	(1.408)	(1.378)
Observations	7,969	7,942	5,877	5,852	7,969	7,942	5,877	5,852
R-squared	0.472	0.474	0.489	0.491				
Model	OLS	OLS	OLS	OLS	Tobit	Tobit	Tobit	Tobit
Fix Effects (FE)	Donor, Year	Donor, Year	Donor, Year	Donor, Year	Donor, Year	Donor, Year	Donor, Year	Donor, Year
Cluster	Dyad	Dyad	Dyad	Dyad	Dyad	Dyad	Dyad	Dyad

Notes: Dependent variable is the log of (one plus) health aid disbursement from donor to recipient in year t. All variables except Gavi Graduate, Colony, Civil War, Democracy, U5MR, DTP3 coverage and HDI are measured in natural logs. *p<0.10, **p<0.05, ***p<0.01.

Table S4.3 Impact of Gavi transition on different types of bilateral ODA and country vulnerability

Variable	All sector ODA M13	Health ODA M14	All sector ODA M15	Health ODA M16	All sector ODA M17	Health ODA M18	All sector ODA M19	Health ODA M20	All sector ODA M21	Health ODA M22	All sector ODA M23	Health ODA M24	All sector ODA M25	Health ODA M26
Gavi graduate	-0.563	-0.525	-1.262	-1.227	-0.227	-0.347	-0.217	0.028	-0.457***	-0.511***	-0.246	-0.423**	-0.158	-0.299
	(1.715)	(1.730)	(1.204)	(1.288)	(0.180)	(0.218)	(0.624)	(0.624)	(0.135)	(0.146)	(0.163)	(0.179)	(0.192)	(0.236)
**Interactions**														
Gavi graduate * distance	0.050	0.078	0.105	0.089										
	(0.188)	(0.180)	(0.141)	(0.150)										
Gavi graduate * donor imports	0.152	0.159			-0.021	-0.017								
	(0.106)	(0.130)			(0.029)	(0.030)								
Gavi graduate * migrants	0.002	-0.037					-0.013	-0.040						
	(0.053)	(0.059)					(0.054)	(0.053)						
Gavi graduate * colony	0.255	0.055							0.358**	0.171				
	(0.217)	(0.250)							(0.179)	(0.208)				
Gavi graduate * US military	-0.010	0.029									-0.071	-0.027		
	(0.070)	(0.069)									(0.065)	(0.067)		
Gavi graduate * donor exports	-0.196*	-0.192											-0.031	-0.0240
**Recipient-donor relationship variables**														
Distance	-0.609***	-0.462***	-0.609***	-0.460***	-0.585***	-0.442***	-0.589***	-0.446***	-0.599***	-0.448***	-0.594***	-0.445***	-0.585***	-0.442***
	(0.107)	(0.103)	(0.107)	(0.102)	(0.101)	(0.096)	(0.102)	(0.096)	(0.102)	(0.097)	(0.103)	(0.097)	(0.101)	(0.096)
Donor imports	-0.021	0.019	-0.018	0.020	-0.018	0.020	-0.018	0.021	-0.020	0.019	-0.018	0.012	-0.019	0.02
	(0.024)	(0.024)	(0.023)	(0.024)	(0.023)	(0.024)	(0.023)	(0.024)	(0.023)	(0.024)	(0.023)	(0.024)	(0.023)	(0.024)
Migrants	0.030	0.043*	0.034	0.044*	0.033	0.042*	0.035	0.045*	0.032	0.043*	0.034	0.044*	0.032	0.042*
	(0.024)	(0.025)	(0.023)	(0.024)	(0.024)	(0.024)	(0.024)	(0.024)	(0.024)	(0.024)	(0.023)	(0.024)	(0.024)	(0.024)
Colony	0.235**	0.094	0.247***	0.099	0.240***	0.0927	0.244***	0.096	0.228**	0.089	0.252***	0.099	0.239***	0.092
	(0.095)	(0.097)	(0.093)	(0.094)	(0.092)	(0.094)	(0.092)	(0.093)	(0.092)	(0.094)	(0.092)	(0.094)	(0.092)	(0.094)
US military	0.114***	0.030	0.116***	0.0322	0.114***	0.031	0.115***	0.031	0.114***	0.031	0.119***	0.033	0.113***	0.030
	(0.023)	(0.023)	(0.022)	(0.022)	(0.023)	(0.022)	(0.022)	(0.022)	(0.022)	(0.022)	(0.022)	(0.022)	(0.023)	(0.022)
Donor exports	-0.017	-0.046*	-0.027	-0.051*	-0.021	-0.047*	-0.025	-0.050*	-0.024	-0.050*	-0.026	-0.051*	-0.019	-0.046*
	(0.028)	(0.027)	(0.026)	(0.026)	(0.027)	(0.026)	(0.026)	(0.026)	(0.027)	(0.027)	(0.026)	(0.026)	(0.027)	(0.027)
**Recipient variables**														
Population	0.427***	0.379***	0.420***	0.375***	0.423***	0.377***	0.420***	0.376***	0.426***	0.377***	0.419***	0.374***	0.424***	0.378***
	(0.036)	(0.036)	(0.035)	(0.035)	(0.036)	(0.036)	(0.035)	(0.035)	(0.036)	(0.035)	(0.035)	(0.035)	(0.035)	(0.036)
GDP per capita	-0.378***	-0.498***	-0.383***	-0.488***	-0.385***	-0.489***	-0.386***	-0.501***	-0.372***	-0.481***	-0.387***	-0.488***	-0.386***	-0.489***
	(0.082)	(0.085)	(0.078)	(0.078)	(0.078)	(0.077)	(0.082)	(0.083)	(0.078)	(0.078)	(0.078)	(0.078)	(0.078)	(0.077)
Disaster	0.022***	0.007*	0.024***	0.007*	0.024***	0.008*	0.024***	0.008*	0.023***	0.007*	0.023***	0.007*	0.0235***	0.007*
	(0.004)	(0.004)	(0.004)	(0.004)	(0.004)	(0.004)	(0.004)	(0.004)	(0.004)	(0.004)	(0.004)	(0.004)	(0.004)	(0.004)
Civil war	0.134*	-0.043	0.139*	-0.038	0.135*	-0.042	0.139*	-0.040	0.136*	-0.040	0.141*	-0.0375	0.134*	-0.043
	(0.073)	(0.072)	(0.073)	(0.072)	(0.073)	(0.072)	(0.073)	(0.072)	(0.073)	(0.072)	(0.073)	(0.073)	(0.073)	(0.072)

Democracy	-0.050**	-0.072***	-0.051**	-0.075***	-0.048**	-0.072***	-0.050**	-0.073***	-0.049**	-0.073***	-0.053**	-0.075***	-0.048**	-0.072***
	(0.021)	(0.020)	(0.021)	(0.020)	(0.020)	(0.019)	(0.020)	(0.019)	(0.021)	(0.020)	(0.021)	(0.020)	(0.020)	(0.019)
U5mr	-0.005***	-0.002	-0.005**	-0.002	-0.005***	-0.002	-0.005***	-0.002	-0.005***	-0.002	-0.005***	-0.002	-0.005***	-0.002
	(0.001)	(0.002)	(0.002)	(0.002)	(0.002)	(0.002)	(0.002)	(0.002)	(0.002)	(0.002)	(0.002)	(0.002)	(0.002)	(0.002)
Dtp3 coverage	0.000	0.006**	0.0001	0.006***	0.001	0.007***	0.001	0.006***	0.001	0.006***	0.000	0.006**	0.001	0.007***
	(0.003)	(0.003)	(0.002)	(0.002)	(0.002)	(0.002)	(0.002)	(0.002)	(0.002)	(0.002)	(0.003)	(0.003)	(0.002)	(0.002)
HDI	-0.733	-1.000	-0.662	-1.075*	-0.617	-1.044*	-0.619	-0.944	-0.720	-1.108*	-0.678	-1.083*	-0.614	-1.043*
	(0.610)	(0.642)	(0.585)	(0.585)	(0.585)	(0.580)	(0.605)	(0.629)	(0.586)	(0.586)	(0.583)	(0.584)	(0.585)	(0.580)
Year	0.427***	0.379***	0.420***	0.375***	0.423***	0.377***	0.420***	0.376***	0.426***	0.377***	0.419***	0.374***	0.424***	0.378***
	(0.036)	(0.036)	(0.035)	(0.035)	(0.035)	(0.036)	(0.035)	(0.035)	(0.036)	(0.035)	(0.035)	(0.035)	(0.035)	(0.036)
Constant	-0.378***	-0.498***	-0.383***	-0.488***	-0.385***	-0.489***	-0.386***	-0.501***	-0.372***	-0.481***	-0.387***	-0.488***	-0.386***	-0.489***
	(0.082)	(0.085)	(0.078)	(0.078)	(0.078)	(0.077)	(0.082)	(0.083)	(0.078)	(0.078)	(0.078)	(0.078)	(0.078)	(0.077)
Observations	10,275	10,275	10,275	10,275	10,275	10,275	10,275	10,275	10,275	10,275	10,275	10,275	10,275	10,275
R-squared	0.463		0.462		0.462		0.462		0.462		0.462		0.462	
Model	OLS	Tobit	OLS	Tobit	OLS	Tobit	OLS	Tobit	OLS	Tobit	OLS	Tobit	OLS	Tobit
FE	Donor, Year	Donor, Year	Donor, Year	Donor, Year	Donor, Year	Donor, Year	Donor, Year	Donor, Year	Donor, Year	Donor, Year	Donor, Year	Donor, Year	Donor, Year	Donor, Year
Cluster	Dyad	Dyad	Dyad	Dyad	Dyad	Dyad	Dyad	Dyad	Dyad	Dyad	Dyad	Dyad	Dyad	Dyad

Notes: Dependent variable is the log of (one plus) health aid disbursement from donor to recipient in year t. All variables except Gavi Graduate, Colony, Civil War, Democracy, U5MR, DTP3 coverage and HDI are measured in natural logs. *p<0.10, **p<0.05, ***p<0.01.
