# Supplementary material for: Strategic donor behaviour and country vulnerability in health aid transitions
Source: BMJ Glob Health. 2023 Nov 8;8(11):e012953. doi: 10.1136/bmjgh-2023-012953 (PMC10632813; doi:10.1136/bmjgh-2023-012953)
Supplement: Supplementary data [file bmjgh-2023-012953supp003.pdf]

Appendix 3 Additional analysis on four donors

Table S3.1 Impact of Gavi transition and other factors on all sector ODA from specific donors

Variable	US (M27)	US (M28)	UK (M29)	UK0 (M30)	JP (M31)	JP (M32)	BL (M33)	BL (M34)
**Gavi graduate**	-0.586*		0.0689		-0.883**		0.207	
	(0.341)		(0.294)		(0.343)		(0.235)	
**Gavi disbursement**		0.309***		0.227**		0.237***		0.0485
		(0.0959)		(0.0992)		(0.0825)		(0.0783)
**Donor-recipient relationship variables**								
Distance	-0.506	-0.583*	0.402	0.393	-1.375***	-1.408***	0.00896	-0.0266
	(0.321)	(0.320)	(0.416)	(0.405)	(0.198)	(0.210)	(0.277)	(0.271)
Donor imports	0.113*	0.114*	0.0776	0.0615	-0.0670	-0.0535	-0.134*	-0.143*
	(0.0633)	(0.0650)	(0.0879)	(0.0840)	(0.0452)	(0.0494)	(0.0778)	(0.0769)
Migrants	0.0453	0.0280	0.0776	0.0667	0.00847	0.00861	-0.0708	-0.0704
	(0.0835)	(0.0836)	(0.0949)	(0.0927)	(0.0464)	(0.0538)	(0.0804)	(0.0809)
Colony	-0.177	-0.188	-0.337	-0.415	0.389**	0.418**	0.998***	0.971***
	(0.266)	(0.251)	(0.329)	(0.306)	(0.188)	(0.197)	(0.273)	(0.274)
US military	0.361***	0.323***	0.203***	0.167***	0.117**	0.101**	0.0407	0.0292
	(0.0650)	(0.0587)	(0.0511)	(0.0531)	(0.0470)	(0.0493)	(0.0504)	(0.0518)
Donor exports	-0.210***	-0.194**	-0.200**	-0.171*	0.0713	0.0651	0.0642	0.0767
	(0.0790)	(0.0791)	(0.100)	(0.0972)	(0.0586)	(0.0630)	(0.0832)	(0.0835)
**Recipient variables**								
Population	0.634***	0.507***	0.801***	0.710***	0.518***	0.402***	0.384***	0.371***
	(0.123)	(0.113)	(0.132)	(0.116)	(0.0554)	(0.0858)	(0.0800)	(0.0788)
GDP per capita	-0.411**	-0.410**	-0.814**	-0.709**	-0.182	-0.250	-0.886***	-0.859***
	(0.192)	(0.191)	(0.310)	(0.285)	(0.146)	(0.185)	(0.259)	(0.258)
Disaster	0.00864	0.00408	-0.00662	-0.00783	0.0164*	0.0132	-0.00357	-0.00293
	(0.0158)	(0.0157)	(0.0187)	(0.0192)	(0.00951)	(0.00985)	(0.0115)	(0.0113)
Civil war	0.239	0.173	1.074***	0.978***	0.186	0.143	-0.0436	-0.0844
	(0.269)	(0.245)	(0.342)	(0.335)	(0.134)	(0.141)	(0.237)	(0.244)
Democracy	-0.120*	-0.108	-0.180**	-0.167**	-0.121***	-0.108**	-0.0341	-0.0279
	(0.0708)	(0.0668)	(0.0758)	(0.0753)	(0.0399)	(0.0438)	(0.0604)	(0.0606)
U5mr	-0.000657	-0.00102	0.000856	0.000220	4.42e-05	0.000801	-0.00678	-0.00717
	(0.00452)	(0.00418)	(0.00830)	(0.00842)	(0.00273)	(0.00293)	(0.00611)	(0.00595)
Dtp3 coverage	-0.00399	-0.00639	0.0197*	0.0178	0.00510	0.00413	0.00294	0.00202
	(0.00649)	(0.00594)	(0.0111)	(0.0114)	(0.00402)	(0.00467)	(0.00734)	(0.00761)
HDI	-0.909	0.186	-0.673	-0.194	0.706	1.737*	0.956	1.082
	(1.342)	(1.312)	(1.830)	(1.618)	(0.865)	(0.889)	(1.816)	(1.835)
Constant	11.64***	11.52***	2.860	2.152	15.32***	15.06***	7.666**	8.065**
	(3.763)	(3.883)	(5.204)	(5.055)	(2.047)	(2.267)	(3.273)	(3.288)
Observations	697	695	635	633	706	704	583	582
R-squared	0.629	0.646	0.600	0.605	0.685	0.681	0.426	0.425
Model	OLS	OLS	OLS	OLS	OLS	OLS	OLS	OLS
FE	Year	Year	Year	Year	Year	Year	Year	Year
Cluster	Dyad	Dyad	Dyad	Dyad	Dyad	Dyad	Dyad	Dyad

Notes: Dependent variable is the log of (one plus) all sector aid disbursement from donor to recipient in year t. All variables except Gavi Graduate, Colony, Civil War, Democracy, U5MR, DTP3 coverage and HDI are measured in natural logs. *p<0.10, **p<0.05, ***p<0.01.

Table S3.2 Impact of Gavi transition and other factors on health ODA from specific donors

**Variable**	**US** (M35)	**US** (M36)	**UK** (M37)	**UK0** (M38)	**JP** (M39)	**JP** (M40)	**BL** (M41)	**BL** (M42)
**Gavi graduate**	-1.216**		-0.602		-0.328*		0.0113	
	(0.545)		(0.515)		(0.176)		(0.300)	
**Gavi disbursement**		0.474***		0.258**		0.151***		0.0310
		(0.134)		(0.125)		(0.0435)		(0.0697)
**Donor-recipient relationship variables**								
Distance	0.0712	-0.0248	0.637	0.693	-0.572***	-0.607***	0.230	0.231
	(0.446)	(0.459)	(0.520)	(0.513)	(0.154)	(0.150)	(0.334)	(0.330)
Donor imports	0.170	0.177	0.293**	0.279**	0.00718	0.00932	-0.148	-0.150*
	(0.113)	(0.117)	(0.128)	(0.123)	(0.0419)	(0.0393)	(0.0910)	(0.0911)
Migrants	0.140	0.116	0.185	0.173	0.0669**	0.0666**	-0.0849	-0.0860
	(0.116)	(0.112)	(0.133)	(0.129)	(0.0340)	(0.0329)	(0.0892)	(0.0891)
Colony	-0.314	-0.299	-0.402	-0.442	0.301**	0.294**	0.622**	0.610**
	(0.361)	(0.336)	(0.391)	(0.380)	(0.139)	(0.136)	(0.312)	(0.309)
US military	0.155*	0.105	-0.00996	-0.0402	0.147***	0.131***	-0.0295	-0.0347
	(0.0794)	(0.0759)	(0.108)	(0.105)	(0.0451)	(0.0438)	(0.0482)	(0.0492)
Donor exports	-0.124	-0.110	-0.284*	-0.259*	-0.0263	-0.0224	0.0437	0.0483
	(0.123)	(0.125)	(0.158)	(0.153)	(0.0447)	(0.0427)	(0.0944)	(0.0948)
**Recipient variables**								
Population	0.668***	0.457***	0.875***	0.752***	0.181***	0.112*	0.386***	0.374***
	(0.151)	(0.141)	(0.172)	(0.160)	(0.0584)	(0.0608)	(0.0913)	(0.0922)
GDP per capita	-0.678**	-0.725**	-1.138***	-1.103***	-0.143	-0.144	-0.740**	-0.734**
	(0.298)	(0.294)	(0.400)	(0.371)	(0.110)	(0.106)	(0.363)	(0.366)
Disaster	-0.0250	-0.0311	-0.0352	-0.0386*	0.0246***	0.0226***	0.00619	0.00624
	(0.0205)	(0.0201)	(0.0225)	(0.0224)	(0.00806)	(0.00803)	(0.0135)	(0.0134)
Civil war	-0.0848	-0.177	0.935**	0.875**	0.0497	0.0151	-0.158	-0.174
	(0.327)	(0.307)	(0.372)	(0.378)	(0.162)	(0.160)	(0.249)	(0.251)
Democracy	-0.259***	-0.240***	-0.337***	-0.324***	-0.0379	-0.0324	-0.0295	-0.0259
	(0.0835)	(0.0780)	(0.0965)	(0.0948)	(0.0448)	(0.0424)	(0.0578)	(0.0574)
U5mr	0.00217	0.00203	0.00399	0.00488	0.000895	0.00118	-0.00414	-0.00402
	(0.00708)	(0.00660)	(0.00908)	(0.00919)	(0.00267)	(0.00263)	(0.00636)	(0.00641)
Dtp3 coverage	0.00862	0.00519	0.0320**	0.0310**	0.00411	0.00324	0.0141	0.0139
	(0.0118)	(0.0112)	(0.0124)	(0.0126)	(0.00409)	(0.00393)	(0.00884)	(0.00894)
HDI	-2.438	-0.533	-3.567*	-2.407	-0.761	-0.243	1.141	1.306
	(1.699)	(1.586)	(1.923)	(2.033)	(0.932)	(0.885)	(3.199)	(3.333)
Constant	6.134	6.187	0.695	-0.857	6.100***	6.015***	2.527	2.357
	(5.779)	(6.010)	(6.327)	(6.296)	(1.817)	(1.818)	(3.646)	(3.650)
Observations	697	695	635	633	706	704	583	582
Model	Tobit	Tobit	Tobit	Tobit	Tobit	Tobit	Tobit	Tobit
FE	Year	Year	Year	Year	Year	Year	Year	Year
Cluster	Dyad	Dyad	Dyad	Dyad	Dyad	Dyad	Dyad	Dyad

Notes: Dependent variable is the log of (one plus) health aid disbursement from donor to recipient in year t. All variables except Gavi Graduate, Colony, Civil War, Democracy, U5MR, DTP3 coverage and HDI are measured in natural logs. *p<0.10, **p<0.05, ***p<0.01.

Mao W, *et al.* *BMJ Glob Health* 2023; 8:e012953. doi: 10.1136/bmjgh-2023-012953
